# Early ocular surface and tear film status in congenital aniridia indicates a supportive treatment window

**DOI:** 10.1136/bjo-2021-320774

**Published:** 2022-12-14

**Authors:** Fabian N Fries, Kayed Moslemani, Tor Paaske Utheim, Berthold Seitz, Barbara Käsmann-Kellner, Neil S Lagali

**Affiliations:** 1 Department of Ophthalmology, Saarland University Hospital and Saarland University Faculty of Medicine, Homburg, Germany; 2 Dr. Rolf M. Schwiete Center for Limbal Stem Cell and Aniridia Research, Saarland University, Homburg, Saar, Germany; 3 Department of Medical Biochemistry, Oslo University Hospital, Oslo, Norway; 4 Department of Ophthalmology, Sørlandet Hospital Arendal, Arendal, Norway; 5 Department of Biomedical and Clinical Sciences, Linköping University, Linköping, Sweden

**Keywords:** Cornea, Genetics, Ocular surface, Tears

## Abstract

**Aim:**

To evaluate changes in the ocular surface and tear film with age and mutational status in congenital aniridia.

**Methods:**

45 participants with congenital aniridia (89 eyes) in a prospective, cross-sectional study. Whole-exome sequencing identified the causative mutation. Examinations included slit-lamp biomicroscopy, in vivo confocal microscopy, Ocular Surface Disease Index (OSDI) score, blink rate, Schirmer I test, Oxford Staining Score (OSS), tear film break-up time (TFBUT) and Ocular Protection Index (OPI).

**Results:**

There were age-dependent increases in OSDI (β=0.34, 95% CI 0.03 to 0.66; p=0.030), blink rate (β=0.18, 95% CI 0.08 to 0.27; p<0.001) and OSS (β=0.05, 95% CI 0.03 to 0.07; p<0.001) and age-dependent reductions in tear production (β=−0.23, 95% CI −0.43 to 0.02; p=0.029) and TFBUT (β=−0.10, 95% CI −0.17 to –0.04; p<0.001). Perturbed OSDI, OSS, blink rate, tear production and TFBUT were noted after the age of ten and OSDI, OSS, blink rate and TFBUT correlated with deficient corneal nerves and limbal stem cell function. OSDI, blink rate, Schirmer, OSS, TFBUT and OPI were not associated with type of *PAX6* mutation, but OSDI, OSS and blink rate associated with grade of aniridia-associated keratopathy.

**Conclusions:**

Ocular surface damage and dry eye signs appear in congenital aniridia regardless of mutation, appearing after 10 years of age and progressing thereafter. An early treatment window may exist for therapies to protect the ocular surface homoeostasis and limbal function, to possibly delay keratopathy development and progression.

**WHAT IS ALREADY KNOWN ON THIS TOPIC**

- Aniridia-associated keratopathy (AAK) is a progressive condition of bilateral limbal stem cell deficiency and inflammation leading to loss of corneal transparency and visual impairment.

**WHAT THIS STUDY ADDS**

- Ocular surface damage and tear film abnormalities are present in all cases, but only appear after the first decade of life. These features are closely linked to worsening corneal nerve and stem cell status.

**HOW THIS STUDY MIGHT AFFECT RESEARCH, PRACTICE OR POLICY**

- There appears to be an early window for supportive treatment of the ocular surface and tear film in congenital aniridia before clinical signs manifest. Early treatment might delay the progression and severity of AAK.

## Introduction

Congenital aniridia is a rare, pan-ocular disease principally caused by mutations in the *PAX6* gene controlling eye development.^1 2^ One of the most serious and vision-threatening manifestations of aniridia is a progressive loss of corneal transparency accompanied by conjunctivalisation of the cornea, a pathology termed aniridia-associated keratopathy (AAK). Recent studies have begun to shed light on a broad spectrum of alterations in the cornea in AAK, including limbal niche degradation, inflammatory cell invasion, corneal nerve degeneration, epithelial cell transformation and lymphatic vessel invasion.^3–6^ The ocular surface and tear film are also affected in aniridia, with studies also reporting the prevalence of dry eye disease,^7–9^ meibomian gland dysfunction (MGD)^8^ and alterations in the tear film composition^10–12^ in aniridia.

With regard to the ocular surface, in a study examining 15 subjects with aniridia Peral *et al* described reduced tear film break-up time (TFBUT) and increased corneal staining, while subjects self-reported symptoms of dry eye.^12^ In a survey of 99 subjects with aniridia of varying ages, 56% self-reported symptoms of dry eye, with clinical dry eye diagnosis occurring at a mean age of 24 years.^9^ Likewise, in a study with twenty aniridia subjects, Jastaneiah *et al* reported signs of dry eye in over 90% of cases, including pathological TFBUT, reduced tear meniscus and pathological meibomian orifices. In a later study, Landsend *et al* reported in 35 aniridia subjects altered tear film osmolarity, increased ocular surface staining and meibomian gland loss.^8^

A clear pathology is present in aniridia at the ocular surface and has increasingly been related to genotype,^13–16^ given the over 500 unique mutations in the *PAX6* gene^15^ known to cause an aniridia phenotype. The degree to which changes in the ocular surface and tear film status in aniridia are affected by genetic mutation, however, is not fully known, nor is it clear at what age such changes begin to appear. Given the progressive conjunctivalisation and limbal stem cell deficiency in AAK causing severe vision impairment, it is useful to gain a better understanding of factors influencing the protection of the ocular surface, as these could potentially give rise to, or accelerate the progression of AAK. Moreover, understanding the age at which the protective function of the tear film is perturbed can influence the choice and timing of treatment or future preventative strategies. For these reasons, the objective of this study was to examine the age and mutation-dependent changes in the tear film and ocular surface in a cohort of subjects with congenital aniridia.

## Materials and methods

### Participants and examinations

All participants who responded and agreed to participate were included. Participants underwent clinical genetic testing based on blood samples provided to various specialised centres in Germany. Genetic mutations were localised using whole-exome sequencing supplemented with multiplex ligation-dependent probe amplification or next-generation sequencing (NGS) methods. The full details of the genetic analysis and mutational subtypes are given elsewhere.^14^ The clinical examinations were conducted in November 2017 and included slit-lamp biomicroscopy grading of AAK according to a previously published grading scale,^14^ slit-lamp examination of the presence or absence of iris remnants, the 12-question Ocular Surface Disease Index (OSDI) questionnaire, blink rate determination (a single, second observer counted the blinks in 60 s while the participant was interviewed by a physician, once during the consultation), Schirmer I test without anaesthesia, examination of ocular surface damage using the Oxford Staining Score (OSS)^17^ with fluorescein staining and the TFBUT test with fluorescein.^4^ The Ocular Protection Index (OPI) was calculated as the TFBUT divided by the interval between successive blinks (60 s/blink rate).^18^ The overall OSDI score was calculated from the responses recorded on the OSDI questionnaire (as OSDI-A, OSDI-B and OSDI-C parts).^19^ In vivo confocal microscopy (Heidelberg Retinal Tomograph 3 with Rostock Corneal Module, Heidelberg Engineering) was additionally used to assess the density of subbasal epithelial nerves (reported as corneal subbasal nerve fibre length density, CNFL, averaged across both eyes) and the predominant central epithelial phenotype, according to an established protocol.^4^ For correlation analyses, epithelial phenotype was graded in terms of worsening pathology, with corneal epithelial cells assigned a value of 0, mixed corneal-conjunctival cells as 1, and conjunctival cells as 2. Additionally, information on the presence of glaucoma and glaucoma medications taken by participants at the time of examination was obtained from patient charts and at the study visit.

### Statistics

To determine the associations between the clinical parameters (OSDI, blink rate, Schirmer I test, TFBUT, OSS, OPI) and independent variables (*PAX6* mutation type, age, sex, AAK grade, iris status), regression analysis was performed with a single parameter value per participant (average of values measured individually for both eyes), using a generalised linear model to include the effects of the independent covariates. Missing data were handled by excluding the individual from the regression analysis. Correlations were tested using the Pearson correlation test. All statistical analyses were performed using IBM SPSS Statistics V.25, IBM, and a critical alpha value of <0.05 was considered significant.

## Results

### Patients and clinical measures

The subjects examined in this study consisted of 45 individuals (89 eyes, 1 eye was enucleated), with clinically and genetically confirmed aniridia. The participant age ranged from 9 months to 64 years (22.2±17.4 years, mean±SD), and there were 23 children aged <18 years (51.1%). Table 1 lists the subjects’ demographic characteristics. Although not the primary focus of this study, prior ocular surgeries included cataract, strabismus, glaucoma, keratoplasty and enucleation. The detailed surgical procedures of the group have been reported elsewhere.^5^

**Table 1:** Demographic and clinical parameters for the aniridia patients examined

Mutation type	Subject no	Age (year)	Sex	AAK grade	OSDI score	Blink rate	Schirmer (mm)	TFBUT (s)	OSS	OPI	IOP (mm Hg)	CNFL (mm/mm^2^)	Epi phenotype	Partial iris
Non-*PAX6* coding	1	4	M	0	9	18	35	12	0	3.6	14	12.7	co	Y
	2	5	F	1	14	10	29.5	13	0.5	2.2	22.5			
	3	10	F	0.5	18	18	33.5	12	1	3.6	14.5	16.0	co	Y
	4	26	F	1	13	16	19	11	0.5	2.9	22	22.0	co	
	5	34	M	0.5	11	20	19.5		3.5		22	14.1	co	Y
Missense	6	4	M	1.5	14	15	35	16	0	4,0	14	8.6	mix	
	7	10	M	1.5	7	17	35	14.5	0	4.1	14.5	17.9	co	
	8	20	F	1	43	25	17.5	14	1	5.8	19	8.2	co	Y
	9	36	F	3	61	24	22.5	9	2.5	3.6	27	0.0	conj	Y
	10	38	F	1.5	59	13	2.5	3.5	4	0.8	13	4.6	mix	
PTC	11 (1)	1	M	0										Y
	12 (1)	9	M	1	10	15	35	11	0	2.8	16			Y
	13	4	M	2	0	17	35	6	0	1.7	8		co	
	14	4	M	2	6	18			0		19.5			
	15	6	F	2	7	15	35	11	0	2.8	24			
	16	7	F	2	15	19	30	15	1	4.8	12.5	11.3	mix	
	17	8	M	1	5	22	24	5.5	1	2.0	15.5	15.4	co	Y
	18	8	F	1.5	14	15	27	12.5	1	3.1	17			
	19 (2)	11	M	2	14	22	21.5	3.5	3	1.3	15.5	7.2	co	
	20 (2)	12	M	2.5	11	18	27	5.5	3.5	1.7	13.5		mix	Y
	21 (2)	46	F	3.5	48	17	23	2			34	0.0	conj	
	22 (3)	11	M	2	39	22	26.5	6	3.5	2.2	19	0.0	conj	
	23 (3)	42	F	3.5	43	21	35	5	4		25	0.0	conj	Y
	24	13	M	2	20	18	15	8	2.5	2.4	14	17.8	co	Y
	25	20	F	4	34	24	35	8	3.5	3.2	12.5	0.0	conj	Y
	26	28	F	2	45	18	22.5	4.5	3.5	1.4	20	6.4	co	
	27	28	F	3	69	22	11.5	2.5	4	0.9	14	0.0	conj	
	28 (4)	34	M	3	27	28	18	9	2.5	4.2	19	0.0	conj	Y
	29 (4)	64	F	3	45	25		4	2			0.0	conj	
	30	36	M	2.5	36	21	26	6.5	3	2.3	19.5	3.0	mix	
	31	42	F	4	45	25	35	3.5	4.5	1.4	15.5	0.0	conj	Y
	32	46	F	1.5	8	15	19	9	2	2.3	25	13.1	mix	
	33	54	M	2	6	36	26.5	3	4.5	1.8	35	3.4	mix	
	34	57	M	2.5	57	25	6	6	4.5	2.5	19	5.4	conj	Y
	35	16	F	3.5	41	20	3	2	5		22	0.0	conj	
	36	52	F	4	70	28	8				20	0.0	conj	
CTE	37	8	M	2	16	15	25	11	1	2.8	17.5			
	38	18	F	2	20	21	17.5	8.5	2.5	3.0	17	6.0	mix	
	39	18	M	2	27	18	31	13	1.5	3.9	14	12.6	co	Y
	40	51	F	3	77	45	35	4	3	3.0	29	0.0	conj	Y
Chromosomal	41	4	F	2	6							1.6	mix	
	42	11	M	4	53	20			4		19			
	43	11	M	4	78	20		3	3.5	1.0	33			
	44	14	F	3.5	36	26	22.5	4	2.5	1.7	12	0.0	conj	Y
	45	20	F	2.5	0	12	35	8.5	2	1.7	16	0.0	conj	

Data represent average across both eyes.

Note: Participant numbering is consistent with the numbering published elsewhere,^14^ with values in parentheses representing family number. A blank cell indicates missing data.

AAK, aniridia-associated keratopathy; CNFL, corneal subbasal nerve fiber length density; co, corneal epithelium; conj, conjunctival epithelium; CTE, C-terminal extension; Epi phenotype, central corneal epithelial phenotype; F, female; IOP, intraocular pressure; M, male; mix, mixed corneal/conjunctival epithelium; OPI, Ocular Protection Index; OSDI, Ocular Surface Disease Index; OSS, Oxford Staining Score; PTC, premature termination codon; TFBUT, tear film break-up time.

### *PAX6* mutational status

Based on linear regression analysis, *PAX6* mutational status (non-*PAX6* coding, missense, premature termination codon, C-terminal extension or chromosomal mutation) did not have predictive value for any of the ocular surface or tear film parameters examined, except for AAK grade, as previously reported.^14^ Therefore, none of the measured ocular surface or tear film parameter values could be explained by *PAX6* mutation type (see table 1 for overview of clinical parameters in relation to mutational status). All five subjects with chromosomal anomalies had full *PAX6* gene deletion. The detailed clinical genetics results have been reported elsewhere.^14^

### Ocular surface and tear film parameters

**Figure 1:**
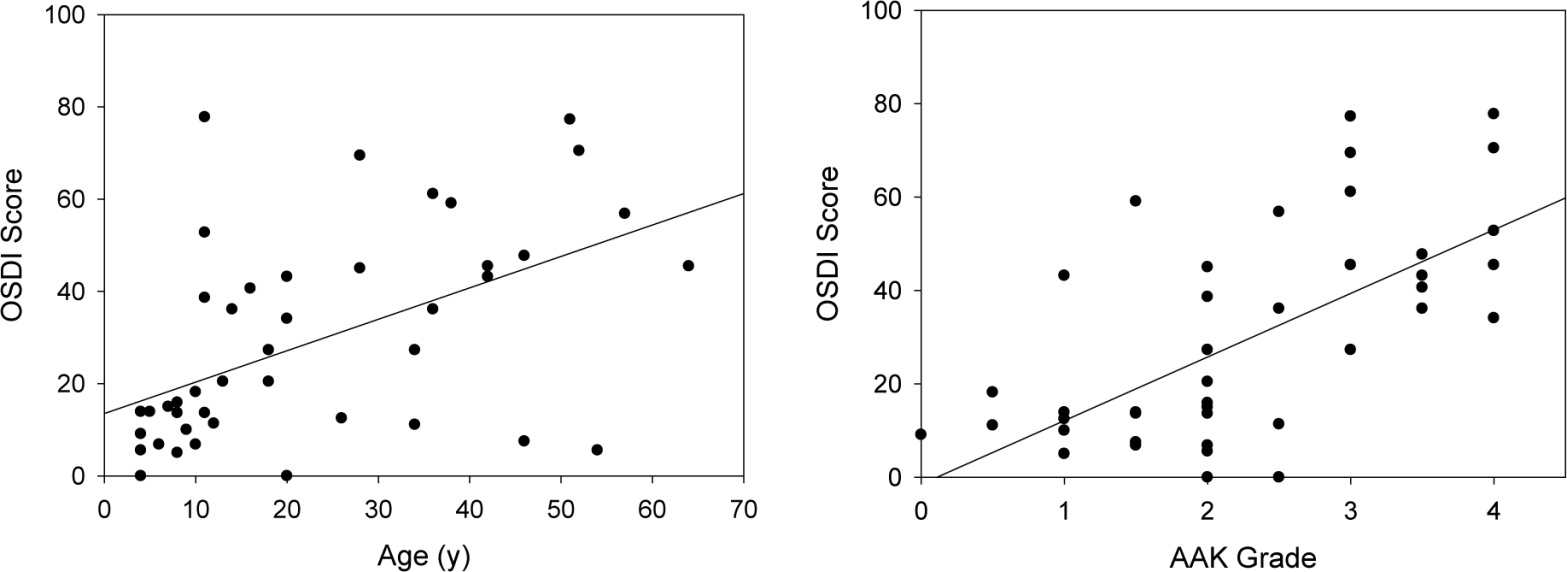
Association of Ocular Surface Disease Index (OSDI) score with age and aniridia-associated keratopathy (AAK) grade in the aniridia patients. The solid line is the linear regression line. The OSDI score correlated with age (p=0.03) and AAK grade (p<0.001).

The overall OSDI score increased with increasing AAK grade and age, controlling for the effects of the other independent variables (figure 1). The OSDI score was strongly correlated with AAK grade (Pearson r=0.634, p<0.001) and age, with each year of life contributing a 0.345 increase to the OSDI score (β=0.345, 95% CI 0.034 to 0.655, p=0.03). Children aged ≤10 years had an OSDI score of <20, while the score increased markedly during the teenage years, generally to >20 throughout life.

**Figure 2:**
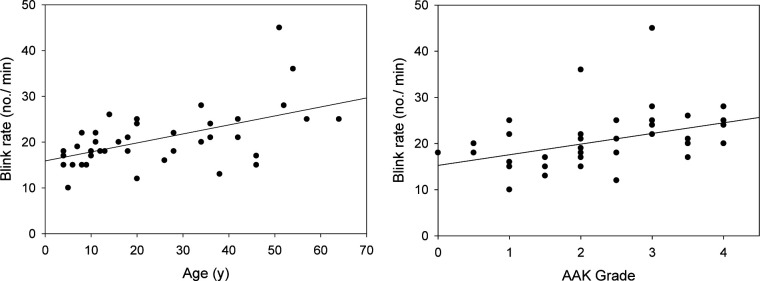
Association of blink rate with age and aniridia-associated keratopathy (AAK) grade in the aniridia patients. Blink rate correlated with age and AAK grade (p<0.001 for both). The solid line is the linear regression line.

The blink rate also increased with age and AAK grade (figure 2). The blink rate was correlated with AAK grade (Pearson’s r=0.547, p<0.001) and age, with each year of life adding 0.18 blinks per minute (β=0.176, 95% CI 0.084 to 0.267, p<0.001).

**Figure 3:**
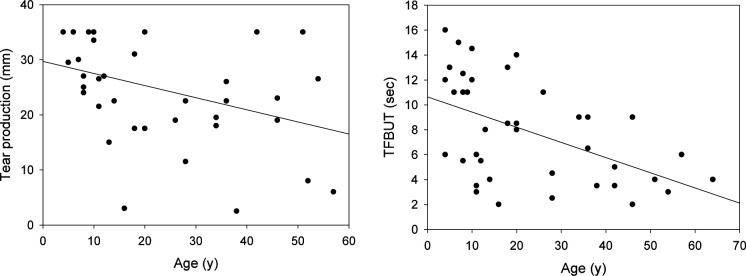
Tear production and tear film break-up time. Left: tear production as measured by the Schirmer I test without anaesthesia. The values reported are the length of wetting of the Schirmer test strip in mm. Tear production declined with age (p=0.029) in aniridia. The solid line indicates the linear regression line. Right: Tear film break-up time versus age in aniridia patients. TFBUT decreased with age in aniridia (p=0.001). The solid line indicates the linear regression line. TFBUT, tear film break-up time.

Tear production, as determined by the Schirmer I test, was associated only with age, with each year of life resulting in a decline of 0.23 mm in wetting of the Schirmer test strip (β=−0.225, 95% CI −0.428 to −0.023, p=0.029). Children aged ≤10 years had high tear production (all over 20 mm/5 min), whereas production tended to decrease in participants aged >10 years; however, tear production among the participants varied substantially (figure 3). Only four participants, however, had a tear production level below 10 mm. The TFBUT decreased with age, with each year of life resulting in a decrease in TFBUT by 0.1 s (β=−0.102, 95% CI −0.165 to −0.039, p=0.001). Additionally, TFBUT was associated with partial vs total aniridia, with complete aniridia resulting in a reduction in TFBUT by 2.4 s relative to the presence of at least a partial iris (β=−2.35, 95% CI −4.53 to −0.169, p=0.035). TFBUT was generally >10 s in children aged ≤10 years, falling to below 10 s in those aged >10 years (figure 3). Notably, all participants over the age of 25 had a TFBUT below 10 s.

**Figure 4:**
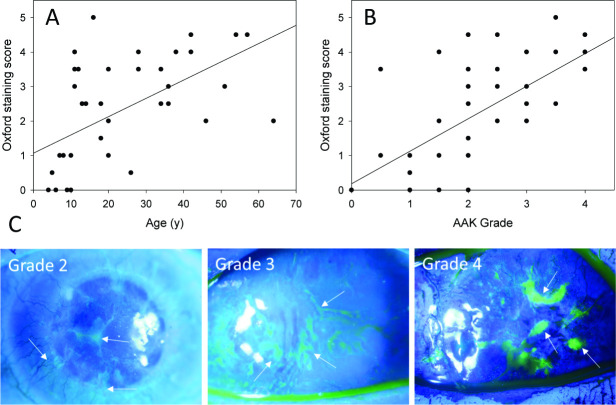
Ocular surface damage assessed by the Oxford Staining Score (OSS) with fluorescein dye. The OSS correlated with (A) age and (B) AAK grade (p<0.001 for both). The solid line is the linear regression line. (C) Ocular surface staining with different grades of AAK. In grade 2 (left panel), vessels have not reached the central cornea, but weak patchy staining is present centrally and peripherally (arrows). In grade 3 (centre panel), islands of staining are apparent throughout the central and peripheral cornea (arrows). In grade 4 (right panel) the ocular surface is opaque and vascularised with areas of strong fluorescein staining (arrows). AAK, aniridia-associated keratopathy.

Ocular surface damage assessed by the OSS increased with age and AAK grade (figure 4A,B). The OSS correlated with AAK grade (Pearson’s r=0.619, p<0.001) and age, with each year of life adding 0.05 to the OSS (β=0.047, 95% CI 0.026 to 0.069, p<0.001). In children aged ≤10 years, the OSS remained at or below 1, while increasing from the teenage years into adulthood, appearing to progress in parallel with AAK progression (figure 4B,C).

The OPI, as a measure of tear film stability, was associated only with the partial presence of an iris. The total absence of an iris resulted in a decrease in the OPI by 1.1 (β=−1.075, 95% CI −2.078 to −0.073, p=0.036).

The CNFL negatively correlated with OSDI score (Pearson’s r=−0.581, p<0.001), blink rate (r=−0.426, p=0.017) and OSS (r=−0.753, p<0.001), while it was positively correlated with TFBUT (r=0.639, p<0.001). A worsening corneal epithelial phenotype positively correlated with OSDI score (r=0.584, p<0.001), blink rate (r=0.371, p=0.026) and OSS (r=0.670, p<0.001), while it negatively correlated with TFBUT (r=−0.606, p<0.001).

Finally, 28 out of 45 participants had glaucoma (prevalence of 62% in this cohort) and were receiving topical IOP-lowering drops at the time of examinations. All topical glaucoma medications were preservative-free formulations, except in one participant (table 1, no. 43 an 11-year-old child with chromosomal deletion but non-WAGR). This participant was subsequently changed to a preservative-free formulation.

## Discussion

We report here in a relatively large and genetically defined aniridia cohort that indicators of ocular surface protection by the tear film and blink reflex are pathologically perturbed in congenital aniridia independent of mutational status. A limitation of the blink rate measurement in this study was that it was manual and thus prone to human error. Ideally in a future study, participants should be video recorded for a more accurate determination of the blink rate. Although tear production measured in this cohort was only pathological (below 5 mm) in three subjects, the basal and reflex tearing were both measured by the Schirmer I test and may have contributed to an overestimation of tear production and a high variability in the measured values. A Schirmer II test, although not performed, may overcome this deficiency. Nevertheless, an age-dependent decrease in tear production was noted along with pathological TFBUT and OSS scores. The condition of the tear film and its integrity appear to progressively deteriorate with increasing age and grade of AAK regardless of the type of *PAX6* mutation causing the aniridia. In the young, the tear production appears normal and relatively unperturbed, and it is during this period that supportive treatment such as artificial tears should be started, to aid in preserving a well-hydrated ocular surface and possibly slow the progression of ocular surface changes.

Insensitivity to the specific *PAX6* mutation suggests that degradation of the tear film and the related ocular surface damage observed may be related to other mutation-independent factors. Interestingly, a deficit in corneal nerves and corneal sensitivity^3–5^ and inflammatory dendritic cell activation^3 5 20^ have also been reported in all aniridia corneas at all ages. The particular cohort reported here also had a corneal nerve deficit and reduced corneal sensitivity, that was more pronounced in older subjects, but with severity that was dependent on whether a *PAX6* coding mutation was present.^5^ The present results are consistent with these prior findings, given the known close relationship between corneal nerves, dendritic cells, dry eye disease and ocular surface homoeostasis.^21–23^ We additionally report here a strong correlation of this nerve deficit with increasing OSDI, blink rate, OSS and decreasing TFBUT, underscoring that the cellular, neural and tear film factors protecting the ocular surface are closely interrelated and secondary to the effect of the mutation, where the primary effect is believed to be dysgenesis of the eye including malformation of the iris and perturbation of the limbal stem cell niche and/or corneal epithelium during development.^24–26^ If this is true, then treating the tear film and nerve deficiencies as well as dampening the inflammatory response to restore a protective ocular surface environment, may be a strategy for supporting the limbal niche function and halting the progression of AAK.^1^ Indeed, the analysis of the limbal stem cell functional status by grading the central corneal epithelial phenotype revealed that a gradual worsening of the epithelial phenotype had the same pattern as the corneal nerve deficit, as epithelial degradation was strongly correlated with increasing OSDI, blink rate and OSS, and decreasing TFBUT. That these parameters were generally normal in the youngest subjects indicates that a potential therapeutic window may exist, before the vicious circle of impaired ocular surface protection, inflammation and limbal degradation causes irreparable damage to the cornea. Thus, a window may be present within the first decade of life, where the ocular surface integrity appears intact, tear production and quality appear to be normal, and symptoms of ocular surface disease are nonexistent to mild, correlating with preserved nerves and limbal stem cell function.

Deterioration in the ocular surface and tear film appeared to start in the teenage years and progress thereafter in the present cohort, consistent with the general progression of AAK with age.^4 5^ Also, more severe iris hypoplasia (total aniridia) was associated with poorer TFBUT and OPI relative to partial aniridia. This may also relate to our previous findings of poorer visual acuity and more advanced AAK grade in those with total aniridia in this cohort.^5^ Thus, when evaluating potential treatments and prognosis for the ocular surface, the degree of iris hypoplasia should be considered.

Many of the participants in the present cohort underwent multiple intraocular surgeries for cataract, glaucoma or other ocular comorbidities, with the number of surgical interventions generally increasing with age in aniridia. These interventions may have impacted the ocular surface and tear film parameters measured in this study and can be considered a limitation of the present analysis. It is only very rarely, however, that an adult with aniridia does not undergo some type of ocular surgery, while children with congenital or early cataract, corneal opacities, glaucoma or other anomalies may also undergo multiple ocular surgeries. This is particularly true for those with chromosomal deletions causing WAGR syndrome, which tends to result in a severe ocular phenotype at a young age.^5 14^

Although not strictly controlled in this study, it is known that children with aniridia may receive various topical therapies as preventative agents or for symptom relief. Use of topical eye medications and compliance in children with aniridia, however, is not well studied. We note, however, that glaucoma medications given to the 62% of subjects in this study with glaucoma were preservative-free with one exception. It is critical to use preservative-free topical formulations given the inflammation present in the cornea and the fragile status of the epithelium in aniridia. From our experience with the early manifestations of aniridia in children and based on the present findings of pathology at the ocular surface in all mutation types with a potential treatment window in the young, we advocate protection of the ocular surface environment in aniridia at the earliest possible age. This can include the use of topical eye ointment of d-panthenol (provitamin B5) in infancy (one application nightly) and starting from the age of 2–3 and onwards, tear replacement drops (water-free semifluorinated alkane formulation EvoTears/NovaTears) during the daytime. This formulation is preservative-free and moistens the ocular surface to stabilise the tears where the lipid layer may not function adequately, in cases of reduced TFBUT and MGD, known to be highly prevalent in aniridia.^8^ Supporting the ocular surface in this way in children may possibly delay the onset of MGD and tear film instability, and in our experience does not induce negative effects in children. In cases of more prominent ocular surface changes, autologous serum eye-drops^27^ or in the case of prominent inflammation, once daily ciclosporin A, could be considered. A low dose of topical ciclosporin A can help to control inflammation and thereby minimise possible damage to corneal nerves and limbal stem cells that long term, persistent inflammation could cause.

Long-term prospective studies with larger genetically defined cohorts are needed to evaluate the generalisability of the results reported here and to evaluate treatment strategies for improving the ocular surface environment in children and adolescents with aniridia. Given, however, the high probability of AAK development and progression, and the difficulties involved in treating AAK, supportive strategies initiated early in life to promote a healthy ocular surface and tear film to potentially delay the onset of ocular surface disease and progression of AAK are warranted.

## Data Availability

All data relevant to the study are included in the article or uploaded as online supplemental information.
